# Characteristics of Pan-Cancer Patients With Ultrahigh Tumor Mutation Burden

**DOI:** 10.3389/fonc.2021.682017

**Published:** 2021-04-22

**Authors:** Hong Yuan, Jun Ji, Min Shi, Yan Shi, Jing Liu, Junwei Wu, Chen Yang, Wenqi Xi, Qingyuan Li, Wei Zhu, Jingjie Li, Xiaoli Gong, Jun Zhang

**Affiliations:** ^1^ Department of Oncology, Ruijin Hospital, Shanghai Jiao Tong University School of Medicine, Shanghai, China; ^2^ Shanghai Institute of Digestive Surgery, Ruijin Hospital, Shanghai Jiao Tong University School of Medicine, Shanghai, China; ^3^ Genecast Biotechnology Co., Ltd, Wuxi, China; ^4^ State Key Laboratory of Oncogenes and Related Genes, Shanghai Jiao Tong University, Shanghai, China

**Keywords:** pan-cancer, hypermutation, tumor mutation burden, mismatch repair, polymerase

## Abstract

**Background:**

Tumor mutation burden has been proven to be a good predictor for the efficacy of immunotherapy, especially in patients with hypermutation. However, most research focused on the analysis of hypermutation in individual tumors, and there is a lack of integrated research on the hypermutation across different cancers. This study aimed to characterize hypermutated patients to distinguish between these patients and non-hypermutated patients.

**Methods:**

A total of 5,980 tumor samples involving 23 types of solid tumors from the in-house database were included in the study. Based on the cutoff value of tumor mutation burden (TMB), all samples were divided into hypermutated or non-hypermutated groups. Microsatellite instability status, PD-L1 expression and other mutation-related indicators were analyzed.

**Results:**

Among the 5,980 tumor samples, 1,164 were selected as samples with hypermutation. Compared with the non-hypermutated group, a significant increase in the mutation rates of DNA mismatch repair genes and polymerase genes was detected in the hypermutated group, and there was an overlap between high TMB and high microsatellite instability or high PD-L1. In addition, we found that EGFR, KRAS and PIK3CA had a high frequency of both single nucleotide variation and copy number variation mutations. These identified mutant genes were enriched in the oncogenic signaling pathway and the DNA damage repair pathway. At the same time, the somatic cell characteristics and distribution of the two groups were significantly different.

**Conclusions:**

This study identified genetic and phenotypic characteristics of hypermutated tumors and demonstrated that DNA damage repair is critically involved in hypermutation.

## Introduction

The fact that many different cancers share common genomic characteristics (1) and respond well to relevant inhibitors has led researchers to perform integrated studies involving multiple types of cancers. Comparison of tumor types analyzed by The Cancer Genome Atlas (TCGA) through the Pan-Cancer Atlas can further supplement and summarize the completed TCGA results (2). The integration of these data sets provides a comprehensive picture of somatic mutations (3, 4), copy number changes (5, 6), mutational signatures (7), and other genetic variations in tumors, furthering the understanding of cancer mechanisms.

Tumor mutation burden (TMB) is defined as the total number of somatic gene coding errors, base substitutions, and gene insertions or deletions detected per million bases (8). The number of somatic mutations in different types of cancers ranges from 0.01 mut/Mb to more than 400 mut/Mb. Tumor antigenicity increases with increased TMB and is a prerequisite for PD1/PDL1 antibody efficacy. In recent years, TMB has been proven to be a good predictor for the efficacy of immunotherapy in multiple clinical trials (9, 10). Retrospective analysis of the CheckMate 568 clinical trial revealed that among patients with advanced/metastatic NSCLC, those with a TMB of 10 mut/Mb or higher had higher objective response and progression-free survival rates than those with a TMB of less than 10 mut/Mb (11). Similar results were observed in the KEYNOTE-028 trial (12).

Hypermutation refers to a cellular mechanism that causes the genome to be mutated at a frequency at least 100,000 to millions of times higher than the background mutation rate. It mainly involves point mutations (single base substitution), as well as occasional base insertion or deletion. Many types of cancers, such as colorectal cancer (13) and gastrointestinal cancer (14,) are classified into two molecular pathological groups: hypermutation and non-hypermutation. Recently, several longitudinal observational studies conducted comparisons of glioma and prostate cancer before and after treatment and found hypermutation differences in the genomes of patients, in particular when the tumor recurs (15–17). In the case of hypermutation, an increasing number of mutations in hypermutant cells may result in decreased fitness, rendering the cells less aggressive and more susceptible to treatment (18). Therefore, hypermutation plays an essential role in tumor occurrence and progression and can improve therapeutic efficacy. However, to date, most research has focused on the analysis of hypermutation in individual tumors, and there is a lack of integrated research on the hypermutation of different cancers.

Here, we performed a comprehensive pan-cancer classification of 5,980 tumor samples involving 23 types of solid tumors from the in-house database (Genecast Biotechnology Co., Ltd). This study aimed to identify the differences in characteristics of the genome mutation profile between patients with hypermutation and those with non-hypermutation (low group). The findings may have significance in guiding clinical practice.

## Materials and Methods

### Genomic and Clinical Data

Genomic and clinical data from 23 different types of solid tumors were gathered from the in-house database (Genecast Biotechnology Co., Ltd). The in-house database was built based on the information collected from clinical samples that was sequenced by a customized 543-gene panel, which covered 1.7 Mb of the genome. The filtering criteria for the samples used in this study were as follows: 1). Samples that were sequenced from January 1, 2019 to December 31, 2019; 2). Samples that were tested by a 543-gene panel; 3). Tissue samples; 4). Patients who aged >25 years old; 5) samples that were collected at the earliest time. The exclusion criteria were as follows: 1). Samples with TMB=0; 2). Tumors with <20 samples; 3). Metastatic samples. The following types of solid tumors were included in the study: non-small cell lung cancer (adenocarcinoma, LUAD, n=2384; squamous cell carcinoma, LUSC, n=456; others, NSCL, n=554), stomach cancer (STAD, n=534), colon cancer (COAD, n=476), rectal cancer (READ, n=344), esophageal carcinoma (ESCA, n=184), liver hepatocellular carcinoma (LIHC, n=162), cholangiocarcinoma (CHOL, n=123), pancreatic cancer (PAAD, n=120), breast cancer (BRCA, n=111), head and neck squamous cell carcinoma (HNSC, n=95), small cell lung cancer (SCL, n=80), ovarian cancer (OV, n=60), cervical squamous cell carcinoma (CESC, n=47), glioblastomas (GBM, n=47), nasopharyngeal cancer (NASO, n=38), skin cutaneous melanoma (SKCM, n=35), bladder cancer (BLCA, n=29), kidney cancer (LICH, n=28), soft tissue sarcoma (SARC, n=28), uterine corpus endometrial carcinoma (UCEC, n=25), and gastrointestinal stromal cancer (GIST, n=20). The cutoff value for hypermutation (8.561943) was determined by the segmented linear regression analysis in R language (19, 20). Among 5,980 Chinese patients, 1,164 (19.5%) had a significantly higher TMB than the others and were identified as patients with hypermutation.

### Identification of Mismatch Repair and Polymerase Gene Mutations

After analyzing the population frequency in the database, as well as the cosmic database and dbSNP database, we screened for nonsynonymous mutations in the exon region or cleavage region of DNA mismatch repair (MMR) genes (MSH2, MLH1, MSH6, and PMS2) and polymerase genes (POL; POLE and POLD1). Manual review was performed to determine the final mutation set. Samples containing more than one mutation in the 6 genes were identified as MMR/POL mutation samples. The Wilcoxon test was used to compare the distribution of TMB between the mutation (MUT) group and wild-type (WT) group, while the difference in the proportion of samples with MUT or WT between the Hypermutation group and Low group was analyzed by using Fisher’s test.

### Analysis of Microsatellite Instability (MSI)

For each microsatellite locus, all spanning reads (covering at least 2 bp in both the 5’ and 3’ directions) were extracted from the realigned BAM file. Following deduplication, the length of the mononucleotide repeat in each deduped alignment was counted and tallied by length. The baseline reference value was calculated by using 30 normal blood samples and was used to assess the instability of microsatellite loci. Finally, the fraction of unstable loci out of the total number of loci analyzed was calculated for each experimental sample. Based on the fraction value, samples were classified into the MSI-H group and MSS/MSI-L group. A fraction value of 0.3 was set as the cutoff value for defining an unstable locus as an MSI-positive locus. The Wilcoxon test was used to compare the distribution of TMB between the MSI-H group and MSS/MSI-L group, while the difference in the proportion of samples with MSI-H or MSS/MSI-L between the Hypermutation group and Low group was analyzed by using Fisher’s test.

### Detection of PD-L1 Expression

The expression of PD-L1 on the surface of tumor cells (TCs) and tumor-infiltrating immune cells (ICs) was assessed by IHC staining using anti-PD-L1 (SP142) rabbit monoclonal primary antibody (Roche, Indianapolis, IL, USA). PD-L1 expression was described as a continuous variable based on the percentage of tumor cells with a certain staining intensity (21). Samples were also classified into the negative (N), low-positive (positive 1, P1), medium-positive (positive 2, P2), and high-positive (positive 3, P3) groups according to the expression level of PD-L1. The Wilcoxon or Kruskal-Wallis test was used to compare the distribution of TMB in the high and low PD-L1 groups, while the difference in PD-L1 expression between the Hypermutation group and the Low group was analyzed by using Fisher’s test.

### Identification of Single Nucleotide Variation (SNV)

Sequencing reads were processed through an in-house pipeline. The pipeline included Trimmomatic (v.0.39) for read adapter trimming and quality filtering, BWA (v.0.7.17) for mapping reads to the hg19 reference genome, the Picard toolkit (v.2.1.0) for sorting and making duplicates, and the Genome Analysis ToolKit (v.3.7) for read realignment. VarDict (v.1.5.1) was introduced for SNV calling, and compound heterozygous mutations were merged with FreeBayes (v.1.2.0). The generated candidate mutations were annotated using the ANNOVAR software tool and then filtered by using the ExAC, COSMIC, and dbSNP databases. Manual curation was performed to generate the final somatic SNV/InDel data set. The differences between the two groups of variation were evaluated by Fisher’s test.

### Identification of Copy Number Variation (CNV)

The GC content, target region length, and read count were corrected. Thereafter, the copy number and gene specificity score (GCS) was calculated using 30 normal blood samples as a control. GCS represents the degree of gene level difference between the tested sample and control. CNV was determined by a joint statistical significance test on GCS and the absolute value of the copy number.

### Pathway and Mutational Signature Analysis

We identified genetic mutations in 10 major cancer pathways and 8 repair pathways in the samples, counted the number of mutations in each pathway for each cancer population and calculated the mutation frequency of each cancer population. The mutational signature was determined based on these somatic SNVs/InDels using maftools (v.2.4.10). The Wilcoxon test was used to compare the distribution and difference of the somatic signature among or between the Hypermutant and Low groups.

## Results

### TMB Screening-Based Detection of Hypermutation

**Figure 1:**
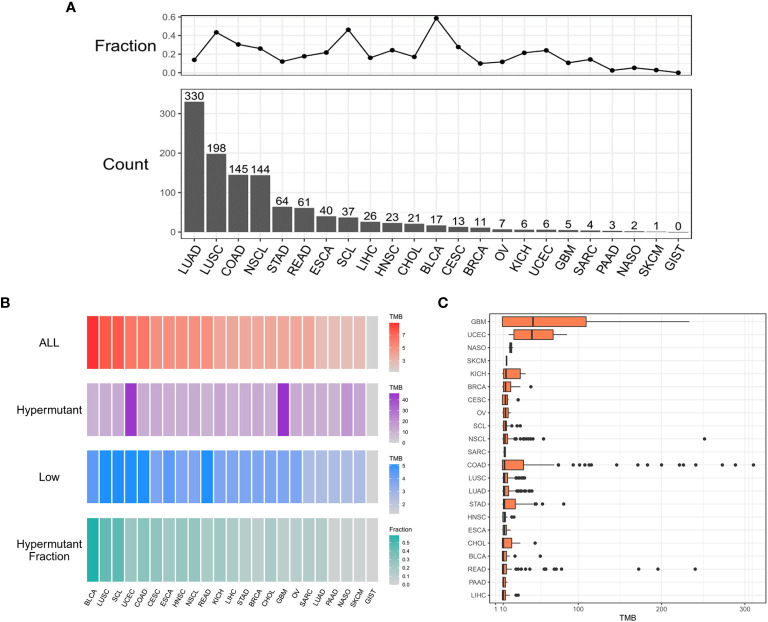
The distribution of cancer types. **(A)** Histogram of the number of samples with hypermutation per cancer type. The numbers were sorted from highest to lowest. The line graph shows the proportion of the number of samples with hypermutation to the total number of samples for each cancer type. **(B)** The median value of TMB in the three sample groups (ALL, Hypermutation, and Low) for each cancer type and the number of Hypermutation samples for each cancer type as a proportion of the total number of samples for that cancer type. **(C)** The distribution of TMB in samples with hypermutation for each cancer type.

As shown in **Figure 1A**, 1,164 patients from a cohort of 5,980 patients with pan-cancer in the in-house dataset were selected as those with hypermutation based on the calculated cutoff value of TMB. The median value of the calculated TMB for each group is shown in **Figure 1B**. Notably, the TMB values of GBM and UCEC were much higher in the Hypermutation group (**Figure 1C**). The age was older and male proportion was higher in the Hypermutation group than in the Low group (p<0.05) (**Table S1**).

### MSI Status and PD-L1 Expression of Patients With Hypermutation

**Figure 2:**
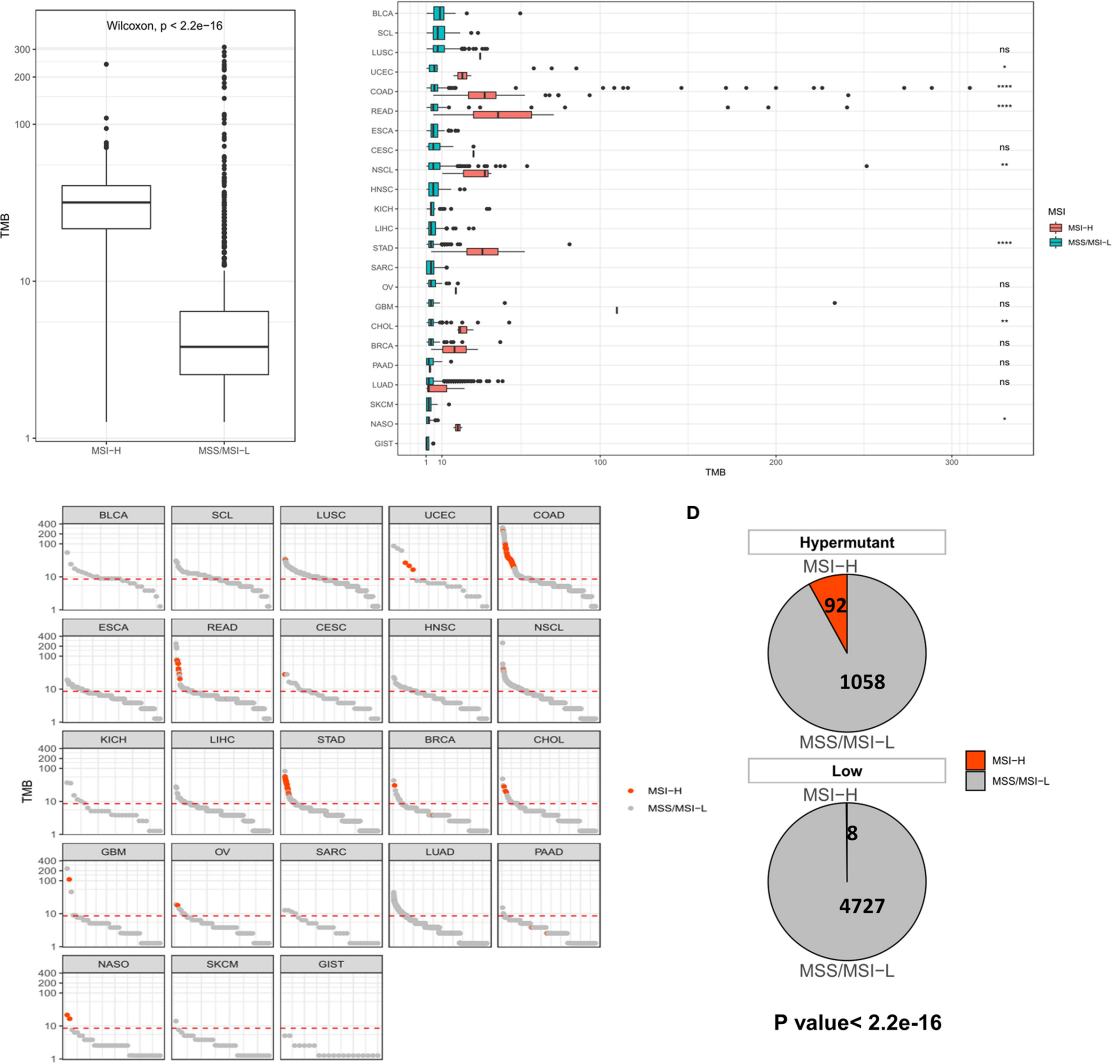
**(A)** Comparison of TMB distribution between MSI-H and MSS/MSI-L groups. **(B)** TMB distribution in different cancer types grouped according to high and low MSI. (**C)** TMB value of each sample in each cancer type. Orange and gray represent MSI-H samples and MSS/MSI-L samples, respectively. **(D)** The number and proportion of MSI-H and MSS/MSI-L samples in Hypermutation and Low groups. *P* <0.05 was considered a significant difference, *P < 0.05, **P < 0.01, ****P < 0.0001, NS, Not Significant.

We next evaluated the impact of TMB on MSI and PD-L1 expression using statistical methods to identify events associated with TMB in solid tumors. MSI, especially high MSI (MSI-H), is closely associated with the occurrence and progression of many tumors. In all samples, the MSI-H samples had a significantly higher TMB than the MSS/MSI-L samples (**Figure 2A**). No difference in TMB was observed between the MSI-H and MSS/MSI-L groups due to the low frequency of MSI in LUSC, HNSC, LICH, PAAD, SKCM, LUAD and other solid tumors. In contrast, there were significant differences in TMB values among UCEC (*P* <0.05), COAD (*P* <0.001), READ (*P* <0.001), NSCL (*P* <0.01), STAD (*P* <0.001), CHOL (*P* <0.01) and NASO (*P* <0.05), indicating that TMB was elevated in MSI-H samples (**Figure 2B**). Moreover, analysis of Hypermutation and Low samples revealed that Hypermutation samples were more prone to MSI-H (**Figures 2C, D**).

**Figure 3:**
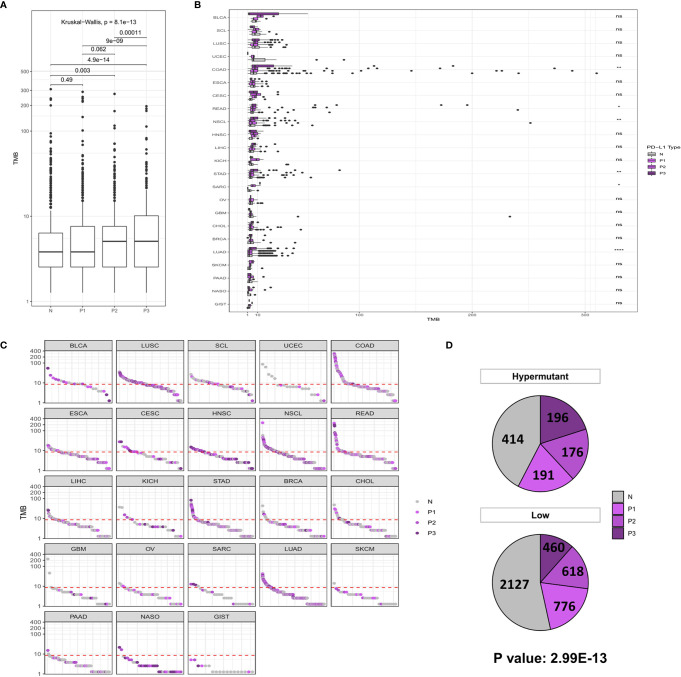
**(A)** Comparison of TMB distribution among N, P1, P2, and P3 groups. **(B)** The TMB distribution of different cancers in the population is shown according to PD-L1 high and low groups. **(C)** TMB value of each sample in each cancer type. Shades of purples indicate the different levels of PD-L1 expression, while grays indicate PD-L1 negative samples. **(D)** The number and proportion of PD-L1 high and low samples in Hypermutation and Low groups. *P* < 0.05 was considered a significant difference, ^*^ *P* < 0.05, ^**^ *P* < 0.01, ^****^ *P* < 0.0001. NS, not significant.

Similar to the analysis of MSI-H characteristics, studies on PD-L1 expression showed that the P2/P3 group displayed a significantly higher overall TMB than the N group, albeit in only six types of tumors, including COAD (*P* <0.01), READ (*P* <0.05), NSCL (*P* <0.01), STAD (*P* <0.01), SARC (*P* <0.05), and LUAD (*P* <0.0001) (**Figures 3A, B**). In addition, a comparison of the difference between Hypermutation and Low samples suggested that there was a correlation between high TMB and high expression of PD-L1 (**Figures 3C, D**).

### Mutational Characteristics of Patients With Hypermutation

**Figure 4:**
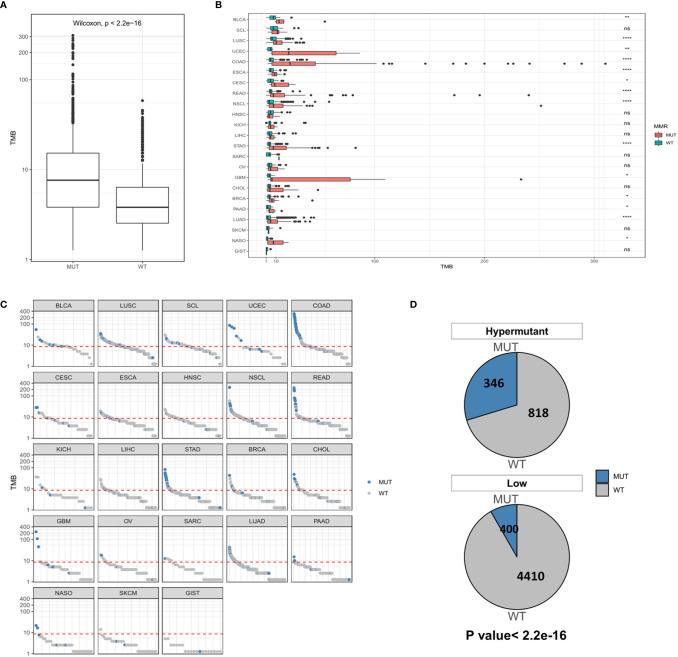
**(A)** Comparison of TMB distribution between the MUT group and WT group. **(B)** The distribution of TMB in the MUT group and WT group for each cancer type. **(C)** TMB value of each sample in each cancer type. Blue and gray represent samples in the MUT group and those in WT group, respectively. **(D)** The number and proportion of MUT and WT samples in Hypermutation and Low groups. *P* < 0.05 was considered a significant difference, ^*^ *P* < 0.05, ^**^ *P* < 0.01, ^****^ *P* < 0.0001. NS, not significant.

While MSI is caused by a defect in MMR genes, POLE or POLD1 mutations serve as immunotherapeutic indicators of all types of tumors except for those with MSI-H. Therefore, we first examined the distribution of TMB in the MUT group and WT group at the global and carcinoma-specific hypermutation level. As shown in **Figures 4A, B**, samples with MMR and/or POL mutations had a higher TMB than the MUT group. The TMB values of COAD, GBM and UCEC were higher than those of other cancer types, and there were significant differences between COAD and UCEC in the MUT and WT groups (*P* <0.0001). Combined with the data from the Low group, a redescription of the mutation landscape for the two types of samples revealed that Hypermutation samples harbored more MMR/POL mutations than the MUT group (**Figures 4C, D**).

**Figure 5:**
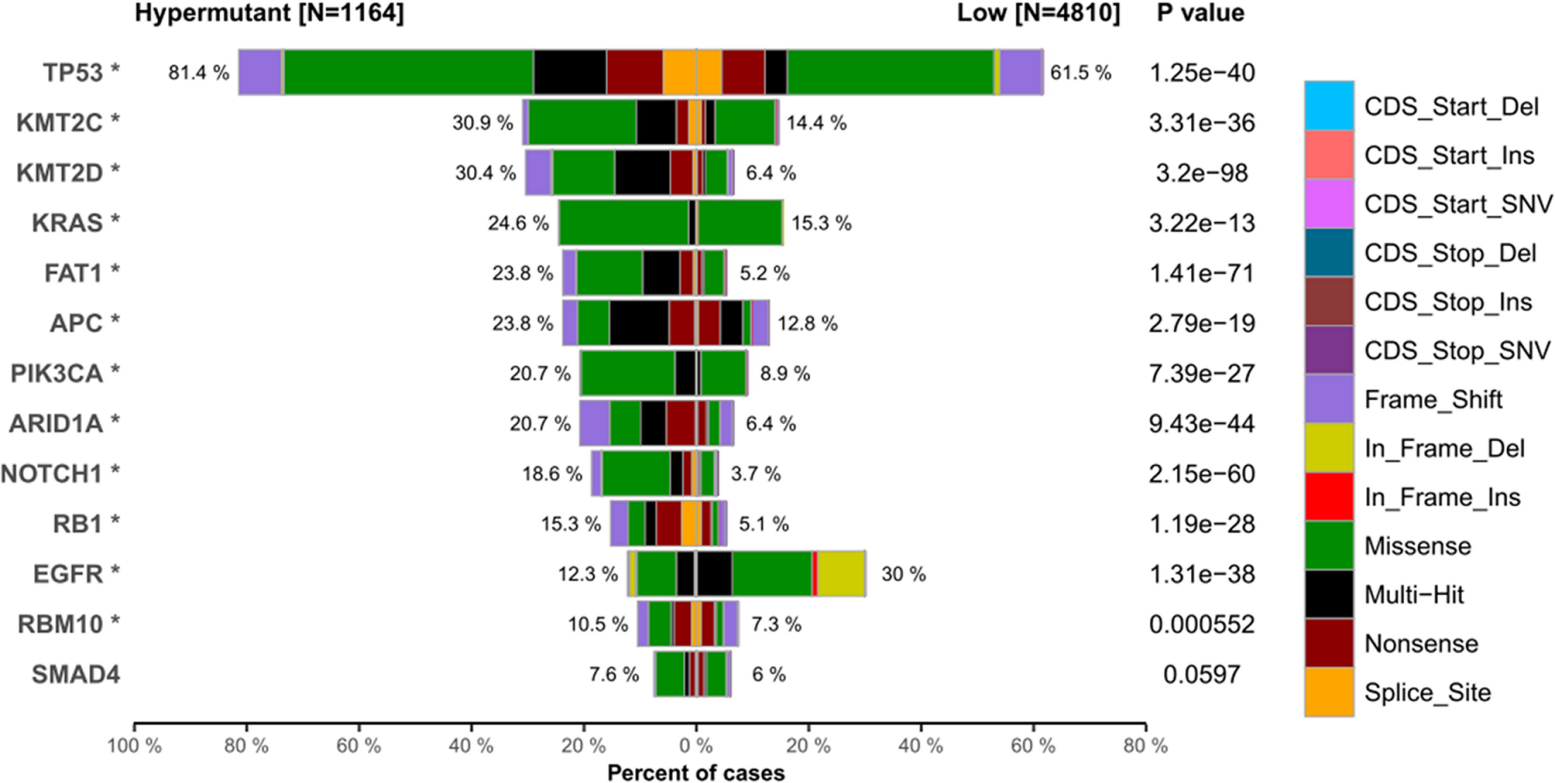
The top 10 high-frequency mutated genes in the Hypermutant and Low groups. ^*^ *P* < 0.05.

We next investigated whether the TMB level affects tumor mutation and CNV burden by quantifying the mutation rate and percentage of CNV in each group. **Table 1** lists CNVs with significant differences between the Hypermutation group and the Low group. Three genes (EGFR, KRAS and PIK3CA) in the top 10 list of mutated genes were identified as having significantly differential CNVs (**Figure 5**). In addition, TP53 was found to be the gene with the highest mutation frequency, with missense mutation as the main mutation type.

**Table 1:** The number and proportion of samples with CNV mutation in the Hypermutation and Low groups.

CNV	Class	Total	Var_num	Var_per (%)	Novar_num	Novar_per (%)	P value	Odds ratio
ALK_GAIN	Hypermutation	633	5	0.79	628	99.21	0.0290771	4.916107
	Low	1857	3	0.16	1854	99.84		
CDK4_GAIN	Hypermutation	633	31	4.90	602	95.10	5.13E-06	0.4267569
	Low	1857	200	10.77	1657	89.23		
EGFR_GAIN	Hypermutation	633	68	10.74	565	89.26	0.001678634	0.642541
	Low	1857	293	15.78	1564	84.22		
KRAS_GAIN	Hypermutation	633	21	3.32	612	96.68	0.02631793	0.5784877
	Low	1857	104	5.60	1753	94.40		
MET_GAIN	Hypermutation	633	21	3.32	612	96.68	0.03308558	0.5904969
	Low	1857	102	5.49	1755	94.51		
PIK3CA_GAIN	Hypermutation	633	103	16.27	530	83.73	5.70E-08	2.148361
	Low	1857	154	8.29	1703	91.71		

Var_num, Number of variation; Var_per, Percentage of variation; Novar_num, Number of no variation; Novar_per, Percentage of no variation; P value and odds ratio were tested using Fisher’s analysis.

**Figure 6:**
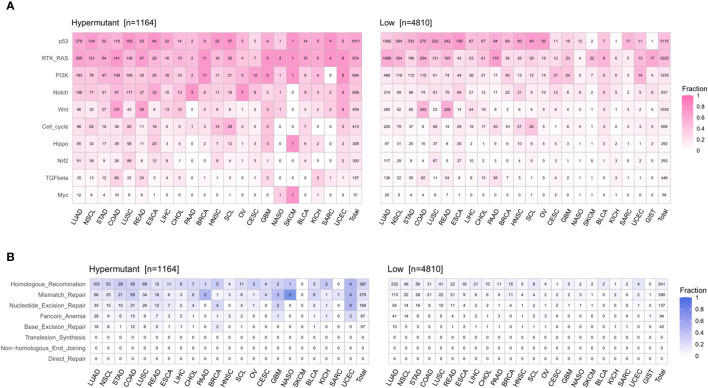
The number and frequency of mutant samples in different pathways in Hypermutant and Low groups for each cancer type. **(A)** The oncogenic signaling pathways were illustrated in pink. **(B)** DNA damage repair pathways were depicted in blue.

**Figure 7:**
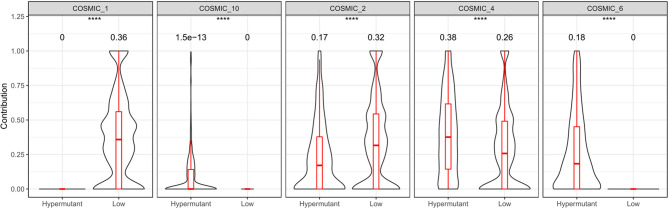
Somatic signature of Hypermutant and Low groups and the proportion of each feature. COSMIC_1: spontaneous deamination of 5-methylcytosine; COSMIC_2: APOBEC Cytidine Deaminase (C>T); COSMIC_4: exposure to tobacco (smoking) mutagens; COSMIC_6: defective DNA mismatch repair; COSMIC_10: defects in polymerase POLE. *P*<0.05 was considered a significant difference, ^****^ *P *< 0.0001.

Statistical analysis of genetic mutations in 10 major cancer pathways and 8 repair pathways in the two types of samples revealed that mutations in the Hypermutation group were mainly enriched in p53 and RTK-RAS cancer-related pathways as well as the homologous recombination and MMR pathways. The mutation frequency of each pathway differed between the two samples (**Figure 6**). To better understand pathways globally dysregulated in the setting of TMB, we further performed a somatic signature analysis in the Hypermutation and Low groups. As shown in **Figure 7**, a total of five signatures, including defective DNA MMR and defects in polymerase POLE, displayed significant differences between the two groups (*P* <0.0001).

## Discussion

In this study, we delineated the distribution of cancer types in patients with hypermutation and identified an association between hypermutation and MSI status, PD-L1 expression and MMR/POL gene mutations. This finding is similar to Gong’s report, which suggested that POLE mutations and MSI tumors (hypermutation phenotype) may increase the expression of immune checkpoint genes, including PD-1, PD-L1 and CTLA-4 (22). Moreover, the present study showed that a comprehensive dissection of high-frequency CNVs, related pathways and somatic signatures, as well as the identification of high-frequency SNVs, are required to identify hypermutation cases with unique characteristics.

Known immune efficacy markers can be roughly divided into two categories: the first is related to tumor neoantigen load, including molecular markers such as MSI or TMB elevation, while the second is related to the tumor T cell inflammatory microenvironment, including core gene markers for PD-L1 protein expression, tumor lymphocyte infiltration and CNV (23). These two types of markers reflect the overall picture of tumor immune efficacy. A combination of two or more methods to determine the immune status of the tumor microenvironment is an effective and universal approach for predicting the efficacy of immune checkpoint inhibitors. In investigating the relationship between MSI or PD-L1 and TMB, we emphasized that the effect of MSI or PD-L1 on TMB mutation rates may vary with tumor type and may be influenced by other endogenous and exogenous tumor factors, such as tumor-infiltrating lymphocytes and microbial flora. It has been documented that a high proportion of hypermutation cooccurs with MSI-H or high PD-L1 expression in colorectal and ovarian cancers (24–26). Interestingly, MMR/POL mutations have been shown to be associated with higher TMB in pan-cancer patients. MSI is caused by MMR defects due to the inactivation of one of the four main MMR genes, MSH2, MLH1, MSH6, and PMS2, and is characterized by extensive polymorphism in microsatellite sequence length as a result of DNA polymerase slippage (27). Furthermore, studies have shown that tumors with MMR defects can also contain other DNA repair defects, such as POLD or POLE mutations, and several immune checkpoint ligands, including PD-1, PD-L1, CTLA-4, LAG-3 and IDO, are also highly expressed in the tumor microenvironment of these patients (28, 29). Therefore, MMR and/or POL mutations may underlie the complex interaction between MSI or PD-L1 expression and TMB.

In the present study, we further demonstrated that patients with hypermutation had a much higher frequency of MMR and/or POL mutations than those with non-hypermutation. On the one hand, we observed that among the eight pathways of the DNA damage response system, the homologous recombination and MMR pathways were the most frequently mutated in the tumor samples. Notably, the correlation between MMR and homologous recombination pathways has been reported in colon cancer and rectal cancer (30). On the other hand, we showed in the somatic signature that hypermutant tumors have defects in both MMR genes and the POLE polymerase gene. Similarly, one study looked at TCGA PanCancer studies involving 10,967 samples as of November 2018 and found 92 POLE exonuclease domain mutations in hypermutant tumors (31).

A disruption of DNA repair pathways will increase mutagenesis and genome instability, thereby affecting cancer progression and drug resistance (32). Here, we found that somatic SNVs in hypermutant tumors are mainly enriched in the p53 pathway. This observation may be linked to the high frequency of TP53 mutations. In addition, SNV and CNV frequency was found to be high in EGFR, KRAS and PIK3CA. Studies using new technologies such as liquid biopsy and next-generation sequencing have revealed that the mechanism of anti-EGFR treatment resistance involves acquired mutations in the KRAS and EGFR ectodomain (33), and PIK3CA mutations are closely related to KRAS mutations (34). These data characterized tumors involving specific gene mutations.

## Conclusion

In this study, we collected data on 5,980 tumor samples involving 23 types of solid tumors and performed a comprehensive analysis on the relationship between hypermutation and gene mutation, MSI, and PD-L1, as well as its clinical significance and characterized the relationship between genotype and phenotype in hypermutant tumors. The overlap between high TMB and MSI-H or high PD-L1 is most likely attributable to MMR and/or POL mutations. In addition, hypermutant tumors displayed a higher rate of cancerous driver gene changes than tumors with non-hypermutation.

## Data Availability Statement

The datasets presented in this study can be found in online repositories. The names of the repository/repositories and accession number(s) can be found below: https://bigd.big.ac.cn/bioproject/browse/PRJCA004800, PRJCA004800.

## Ethics Statement

The studies involving human participants were reviewed and approved by Ruijin Hospital. The patients/participants provided their written informed consent to participate in this study.

## Author Contributions

JZ conceived the study. HY, JJ, and JZ designed the study and wrote the draft manuscript. MS, YS, JLiu, and JW analyzed and interpreted the data. CY and WX collected and interpreted the clinical data. QL and WZ extracted the data and assess the data quality. JLi and XG did literature searches and identified the eligible studies. All authors contributed to the article and approved the submitted version.

## Funding

The study was supported by National Science Foundation of China (81972707), Shanghai Rising-Star Program (20QA1406200), Shanghai Municipal Commission of Health and Family Planning (20184Y0048), Guangci Distinguished Young Scholars Training Program (GCQN-2018-A06), and Shanghai Anticancer Association EYAS PROJECT (SACA-CY19C19).

## Conflict of Interest

QL, WZ, JL and XG were employed by Genecast Biotechnology Co., Ltd.

The remaining authors declare that the research was conducted in the absence of any commercial or financial relationships that could be construed as a potential conflict of interest.
