# Supplementary material for: Characteristics of Pan-Cancer Patients With Ultrahigh Tumor Mutation Burden
Source: Front Oncol. 2021 Apr 22;11:682017. doi: 10.3389/fonc.2021.682017 (PMC8100597; doi:10.3389/fonc.2021.682017)
Supplement: Supplementary file 1 [file Table_1.docx]

Table S1. Clinical statistics of 5,980 patients.

|  |  | Hypermutation (n=1164) | Low (n=4816) | P-value (Fishser) |
| --- | --- | --- | --- | --- |
| Age |  |  |  | 6.58E-12 |
|  | >=60 | 738 (63.4%) | 2518 (52.3%) |  |
|  | <60 | 426 (36.6%) | 2298 (47.7%) |  |
|  |  |  |  |  |
| Gender |  |  |  | < 2.2e-16 |
|  | Male | 912 (78.4%) | 2680 (55.6%) |  |
|  | Female | 252 (21.6%) | 2136 (44.4%) |  |
